# Determinants of Intentions toward Smoking Hookah in Iranian Adolescents Based on the Theory of Planned Behavior

**Published:** 2019-07

**Authors:** Shahzad PASHAEYPOOR, Reza NEGARANDEH, Nasrin NIKPEYMA, Zahra AMROLLAH MAJD ABADI

**Affiliations:** 1. Department of Community Health and Geriatric Nursing, School of Nursing and Midwifery, Tehran University of Medical Sciences, Tehran, Iran; 2. Nursing and Midwifery Care Research Center, Tehran University of Medical Sciences, Tehran, Iran

**Keywords:** Smoking water pipe, Adolescent, Theory of planned behavior

## Abstract

**Background:**

Smoking hookah is considered a health problem among the adolescents, which currently has a high prevalence. The present study aimed to determine the reasons for tendency toward smoking hookah.

**Methods:**

Participants of this qualitative study were 24 adolescents from Tehran selected using purposeful sampling method in 2017. Data gathered through individual semi-structured interviews and two focus groups. Using directed content analysis approach 423 primary codes were categorized into 28 subcategories and 9 categories.

**Results:**

Most of the participants were 16 to 18 yr old (64%), male (58%) and had high school degree (58%) and 75% of them were student. In Theory of Planned Behavior, attitude toward smoking hookah contained categories of replacement for cigarette, the pleasurableness of hookah, easy access and spending the leisure times. Subjective norms contained the categories of family, friends and society. The construct of behavioral control contained the categories of personal and social factors.

**Conclusion:**

Our Results could be helpful in policy-making and planning toward improving the awareness and changing the attitude and performance of the adolescents toward smoking hookah.

## Introduction

Consumption of tobacco as a global problem is one of the preventable causes of death. According to the report of WHO, until 2030, deaths due to the tobacco-related causes would increase to 8 million people and the rate of increase would be higher in developing countries (1–3).

Hookah also known as the water pipe, shisha, arghile, narghile, hubble-bubble, and Qalyan is a traditional way for tobacco consuming. Smoking hookah (SH) has an increasing rate in the world (4, 5). SH has become a global trend among adolescents (5–9). The increase in SH is more common among the adolescents of the Middle East (9). In this region, SH was also considered conventional and in some countries, it is introduced to the adolescents at the ages of 5 to 18 even by the family members. Therefore, its prevalence reaches up to 9% to 15% in the Eastern Mediterranean countries (10, 11).

During the recent years SH has increased among the Iranian adolescents (12–14) and a study in south of Iran showed that the prevalence of SH in the ages of 18–24 is 4.9 times higher than other ages (14).

SH is associated with many health threats. The smoke of the hookah’s tobacco contains high levels of toxins (6, 7). Moreover, one time of SH would increase the carbon monoxide pollution in the air 5 times more than one cigarette; the blood nicotine level in people who smoke hookah is equal to those who smoke 10 cigarettes per day. The smoke of hookah contains carbon monoxide, polyhydrocarbons, formaldehyde, nitrogen, nitric acid and nicotine (15) and is a serious health threat that would lead to lung cancer, cardiovascular diseases, reduced pulmonary function and nicotine dependency, increased heart rate and blood pressure, nausea and vomiting, tuberculosis, chronic bronchitis, bronchial cancer, atherosclerosis, lip carcinoma, poisoning with carbon monoxide, depression and addiction to nicotine (6). Other diseases such as increased levels of helicobacter pylori, hepatitis A, hepatitis C, herpes and dental diseases have also been reported (7). It could also decrease life expectancy, increase treatment costs and decrease creation in different aspects of the individual’s life (3).

Despite the harms of hookah, its consumption is increasing among Iranian adolescents and it has become a common pleasure among adolescents. Therefore, it is necessary to design and perform more effective interventions, especially for this target group (14, 16, 17).

The adolescence requires attention that is more specific because, during this period, the individual would form their lifestyle and prepares for the youth; so preventive and educational measures are necessary for stopping the trend of consumption in this group (18, 19).

To succeed in changing behavior, health care providers should be aware of its affecting factors (20). The Theory of Planned Behavior (TPB) has been used for predicting different behaviors (16, 21, 22). In TPB intention is considered as the main determinant of the behavior and influenced by three constructs of “attitude towards the behavior”, “subjective norms” and “perceived behavioral control” (23). Since our knowledge of the reasons for the tendency of adolescents to SH is low, TPB was considered as a theoretical framework to gain deep knowledge about this. In such situations, the directed content analysis is well suited. TPB can provide predictions about the variables of interest or about relationships among variables (24, 25). Therefore, the aim of this study was to determine the reasons of tendency towards SH among adolescents based on the TPB.

## Methods

This qualitative study has used directed content analysis approach for analyzing the experiences of Iranian adolescents about SH based on the TPB.

Research participants were 24 adolescents who have rich experiences in SH in 2017. Other inclusion criteria were being 15 to 18 yr old, and willingness to describe their experiences. They were selected using purposeful sampling from traditional coffee shops and entertainment locations in Tehran, Iran. Fourteen in-depth interviews and two focus groups were used for gathering the data. The participants were asked to describe their experiences in SH. Open-ended questions like “would you talk about your experiences about smoking hookah?”, “What do you know about hookah?”, “How do you feel about smoking hookah?”, “Who encourages you?”, and “Do you have control over your behavior?” were used for data collection. The interviews lasted about 20 to 40 min and 4 of the participants were interviewed twice.

Principal investigator conducted all the interviews. The interviews were audiotaped, transcribed verbatim and analyzed using directed content analysis. The aim of the directed content analysis was to accredit and develop the used theory in the research so that the theory would help specify the key concepts or variables as categories (26). Deductive content analysis (directed content analysis) is used when the frame of analysis is operationalized based on previous knowledge (24). When the categories have been developed based on theory, all the data are reviewed for content and coded for correspondence with or exemplification of the identified categories (27). Therefore, pre-specified categories could also be used for data analysis in directed content analysis (24). Consequently, the extracted codes, subcategories and categories from the interviews were placed in the themes or constructs of the TPB. In the present study, 423 primary codes were extracted categorized into 28 subcategory and 9 main categories.

### Trustworthiness

Four criteria of credibility, dependability, transferability and conformability were used to increase the rigor of the study (26). The key strategies are prolonged engagement, peer review or debriefing, reflexivity, member-checking, thick description, and external audits.

### Ethical approval

This study was approved by the Ethics Committee of the Tehran University of Medical Sciences (Code: 26975). The objectives of the study were explained and written consents were obtained from all the participants. As well, recorded and written materials were stored securely.

## Results

Data analysis revealed that the mean age of the participants was 16±1.4 yr. Demographic characteristics of the participants are shown in Table 1. Main results are organized and presented based on three constructs of the TPB (Table 2).

**Table 1:** Socio-demographic characteristics of the participants (n = 24)

***Characteristic***		***N (%)***
Gender	Male	14 (58)
	Female	10 (42)
Age (yr)	13–15	8 (33)
	16–18	16 (64)
Educational Level	Illiterate	0 (0)
	< High school	10 (42)
	High school	14 (58)
Marital Status	Married	0 (0)
	Single	24 (100)
Employment Status	Employed	6 (25)
	Student	18 (75)
	Employed+ Student	8 (33)
Family Income	Less than US$150 per month	4 (17)
	US$150 to US$450 per month	17 (71)
	More than US$450 per month	3 (12)

**Table 2:** Themes, categories and subcategories

***Main theme***	***Categories***	***Subcategories***
Attitude toward smoking hookah	Replacement for cigarette	Fewer harms than cigarette Better social acceptance than cigarette
	Pleasurableness of hookah	Feeling of joy Feeling of comfort Attractiveness of the taste and smell Entertainment Attractiveness for being new
	Easy access	Easy access to hookah Affordability of hookah Existence of traditional restaurants and coffeehouses
	Spending leisure time	Hookah as an entertainment Not having any plans for leisure times
Subjective norms for smoking hookah	Family	Being a part of the family’s traditions Being ordinary in the family Lack of strict confrontation from the family Being used by the peers in the family
	Friends	Pressure and encouragement by the friends Being used in friendly gatherings Willingness for experiencing along with the friends Spending time with friends
	Society	Being ordinary in society Being legal Society’s acceptance
Behavioral control over smoking hookah	Personal factors	Not realizing the need for quitting hookah Not believing in the addictive nature of hookah Not believing in the harmfulness of hookah
	Social factors	The appropriate social context for smoking hookah Peers’ pressure

### 1- Attitude:

Attitude towards behavior is one of the constructs of the TPB, which comprises cognitive information in the form of beliefs about the behavior. Experiences of the participants in this construct were placed into four following categories.

1-1- Replacement for cigarette: Participants believed that hookah has less harm compared to cigarette and is socially more acceptable. They stated:
  “I first smoked, when I quit the cigarette, I was lost something, I was somehow, suggesting my friend to replace the hookah. They say less harm.” (Participant No. 12)
1-2- Pleasurableness of hookah: Participants would feel pleasure and comfort by SH and the taste and smell of hookah are attractive for them. They smoke hookah for entertainment. On the other hand, they were drawn to the freshness of experiencing hookah and were interested in the experience. One of the participants mentioned:
  “I like it a lot because of the sense of pleasure and comfort that it gives me, so whenever I go out with my friends or family, I would take it with me and smoke. I even prepare it at home and smoke because it gives me lots of pleasure and peace.” (Participant No. 3)
1-3- Easy access to hookah: the existence of traditional restaurants and coffee shops has made hookah easily accessible and affordable entertainment. One of them stated:
  “Another reason is that it is available in traditional restaurants with reasonable price .” (Participants No. 6). Another teenager said, “You could smoke a hookah with only 10 thousand Tomans.” (Participants No. 10)
1-4- Spending the leisure time: Participants had no specific plans for spending their leisure time after school or work and considered hookah as entertainment for spending their leisure time.
  Participant No. 5 said: “Then, only where we are, we get together with my friend. Let’s go. Then we’ll take a hookah in all condition: angry, happy and etc. and enjoy it. ”
  “Many of the top students would also go for SH because they have nothing else to do. People might say study or read a book, but even then, whenever we get free time, we would go for smoking hookah.” (Participant No. 7)

### 2- Subjective norms

Another construct of the TPB is subjective norms. This construct evaluates the effect of important key persons in individual’s life in the occurrence of the behavior. This construct contains the main categories of family, friends, and society.

2-1- Family: SH is very common in the families, it is used during traditional ceremonies and its smoking is not prohibited. Families are not strict about SH and consider it normal. SH by the peers in the family is also an important factor for tendency towards smoking hookah. They mentioned:
  “I started smoking many years ago because it is a tradition in our family; even if men do not smoke hookah, women would do so. It is a part of our services for the guests during the funerals and other ceremonies. Not all the kids but girls usually start smoking around the age of 15 or 16.” (Participant No. 5)
2-2- Friends: Peers’ pressure is one of the important factors in starting and continuing smoking of hookah. They were interested in gathering with their friends and smoke hookah in their friendly gatherings. Even many of them did not find it interesting to smoke hookah alone. Participants believed that their interest in experiencing hookah with their friends and their tendency to spend time and have fun with their friends was one of the reasons for their tendency toward SH. Two of the participants stated:
  *“The hookah brings friends together that is why it is so popular in Iran.”* (Participant No. 10)
  *“I have a good feeling toward hookah; I always go with my friends for smoking. Being with my friends makes me want to smoke hookah, if my friends are not with me, I would not smoke hookah.”* (Participant No. 11)
2-3- Society: Study participants also considered society as one of the reasons for their tendency toward hookah. SH is not illegal in society and is considered normal and acceptable by society. They expressed their opinions as:
  *“Hookah surely is bad; they say it harms 70 times more than cigarettes but most of the adolescents and youth have tendency toward it because they do not have any jobs or entertainments. Hookah is both legal and affordable and now is considered a normal pleasure in society. It is easy to smoke hookah in the traditional restaurants, in the parks or in the car and no one would bother you.”* Participant No. 12)

### 3- Perceived Behavioral Control

Perceived behavioral control is another construct of the TPB and indicates whether the individual has any control over their behavior (continuing or quitting the behavior) or not.

3-1- Personal factors: SH is not addictive and does not cause physical dependency and has no harm for them; so they could control their behaviors.
  *“This is not cigarette that could harm you. I believe that something could be bad when it is consumed excessively, I do not smoke that much. I do not believe that it is bad. I just smoke two or three times a week and this is not harmful; I have not felt any harms yet.”* (Participant No. 11)
  *“SH is not addictive, it is harmful, but it does not have dependency; I could quit smoking for a month or even smoke every day.”* (Participant No. 6)
3-2- Social factors: as well, the popularity of SH in the society and the peers’ pressure would prevent them from controlling their behavior.
  *“I think that, even once, for two or three months, I did not smoke hookah, but when I go out with my friends and they are smoking, I cannot stop myself. In fact, I do not believe that it is an addiction, because I might not smoke even for a couple of weeks and nothing would be wrong.”* (Participant No. 10)

## Discussion

Three constructs of attitude, subjective norms and perceived behavioral control were main categories. Findings in the construct of attitude showed that one of the reasons for adolescents’ tendency toward SH is their positive attitude toward hookah. Attitude toward smoking cigarette was the third useful variable for predicting cigarette consumption. In other words, positive attitude toward cigarette would increase the chance of smoking. Moreover, attitude toward hookah was effective in predicting smoking hookah. These results were in line with the results of the present study (28).

Hookah was an appropriate replacement for cigarette. SH was harmless and it has a better image in the society. People’s fallacy about harmlessness of hookah and its higher social acceptance than cigarettes is the reasons for higher prevalence of SH compared to cigarette (29).

However, not only hookah is not less harmful than cigarettes, but it is associated with higher risks for respiratory diseases and cancers (30) and in some cases, its damages are more severe (30–33). Therefore, health communication about the damages of hookah and more pervasive restrictions on SH in public places seems necessary (34).

Adolescents in this study were considered SH as kind of entertainment and used it in their leisure times. The reason for SH was pastime and pleasure (16). SH was a kind of entertainment and a symbol of social interaction for spending the leisure time (35, 36). For that reason, providing substitute recreation activities for adolescents is necessary (34).

Affordability and availability of hookah for adolescents was another reason for adolescents’ tendency toward SH. The context was one of the reasons for tendency toward SH in the city of Bushehr (37). Moreover, a qualitative study in Malaysia was revealed that availability, affordability, pleasant smell and not being addictive were the reasons for tendency toward hookah (38). Some studies also mentioned the growth in the number of hookah cafes as one of the reasons for the increase in the rate of SH (39).

All of the participants responded that they all started SH under the pressure of their friends. The role of friends has also been mentioned in other studies (40, 41). As well, hookah has been a part of the Iranian traditions from a long time ago and families have never considered its threat serious; they always believed that hookah was harmless (42). Social and family factors, especially the pressure by the peers, and having a smoker mother have been intensely related to SH among the adolescents (41). Hence, informing families and society about the harms of hookah and building more restrictions upon SH in public places can substantially reduce its consumption (34).

Hookah is not addictive, and they could control their behavior at any desired time and quit smoking. On the other hand, some believed that until they are being pressured by their friends and hookah is considered acceptable in society, they could not have control over their behavior. Therefore, advising adolescents about SH destructive effects and teaching assertiveness to them can be helpful in mitigating the impact of peer pressure.

This study has some limitations, which have to be pointed out. Participants may feel unwilling to participate in this study or reveal their opinions to the researchers.

## Conclusion

Personal attitude, subjective norms and lack of behavioral control could contribute to adolescents’ behavioral intention for SH. Therefore, theory-based interventions could be helpful to reduce SH behaviors.

## Ethical considerations

Ethical issues (Including plagiarism, informed consent, misconduct, data fabrication and/or falsification, double publication and/or submission, redundancy, etc.) have been completely observed by the authors.
